# Use of oral anticoagulants and its associated factors among nonvalvular atrial fibrillation patients with new‐onset acute ischemic stroke: A report from the China Atrial Fibrillation Registry study

**DOI:** 10.1002/clc.23759

**Published:** 2021-12-24

**Authors:** Jing‐Rong Wang, Xin Du, Jian‐Zeng Dong, San‐Shuai Chang, Chao Jiang, Cai‐Hua Sang, De‐Yong Long, Ri‐Bo Tang, Hai‐Bin Zhang, Jin‐Cheng Guo, Yu‐Mei Wen, Liu He, Chang‐Sheng Ma

**Affiliations:** ^1^ Department of Cardiology National Clinical Research Centre for Cardiovascular Diseases, Beijing Advanced Innovation Center for Big Data‐Based Precision Medicine for Cardiovascular Diseases, Beijing Anzhen Hospital, Capital Medical University Beijing China; ^2^ Department of Cardiology, Cardiovascular Center, Beijing Luhe Hospital Capital Medical University Beijing China

**Keywords:** anticoagulant therapy, atrial fibrillation, stroke

## Abstract

**Background:**

The adherence of oral anticoagulant (OAC) therapy among nonvalvular atrial fibrillation (NVAF) patients with acute ischemic stroke (AIS) in China during recent years was unclear, and the possible factors that influenced the initiation and persistent use of OAC were needed to be explored.

**Methods:**

A total of 1085 NVAF patients, who experienced new‐onset and nonfatal AIS from August 2011 to December 2020 during follow‐ups in the China Atrial Fibrillation Registry (China‐AF), were enrolled. Information including patients' demographic characteristics, medical history, medication usage, which were collected before and after the index stroke, were used in the analysis.

**Results:**

OAC was initiated in 40% (434/1085) NVAF patients within 3 months after new‐onset AIS. High‐reimbursement‐rate insurance coverage (odds ratio [OR]: 1.51, 95% confidence interval [CI]: 1.03–2.22, *p* = .036), 3‐month‐peri‐stroke AF episodes (OR: 2.63, 95% CI: 1.88–3.69, *p* < .001), and pre‐stroke OAC usage (OR: 8.92, 95% CI: 6.01–13.23, *p* < .001), were positively associated with initiation of OAC within 3 months after new‐onset AIS, while age (OR: 0.98, 95% CI: 0.96–1.00, *p* = .024), female (OR: 0.63, 95% CI: 0.44–0.90, *p* = .012) and higher modified HASBLED score (OR: 0.45, 95% CI: 0.37–0.55, *p* < .001) were negatively associated with it. Among 3‐month‐post‐stroke OAC users, history of radiofrequency ablation (hazard ratio: 1.65, 95% CI: 1.16–2.35; *p* = .006) was positively associated with non‐persistence of OAC usage.

**Conclusions:**

In China, the proportion of NVAF patients who initiated OAC therapy since new‐onset AIS was still low. More efforts are needed on improving patients' adherence to anticoagulant therapy.

## 1 INTRODUCTION

Atrial fibrillation (AF)‐related cardioembolic stroke is usually more severe than other types,^1^, ^2^ and nonvalvular atrial fibrillation (NVAF) patients with acute ischemic stroke (AIS) are at high risk of recurrence.^3^, ^4^ It was reported that the cumulative recurrence incidence of stroke was 13.8% at 5 years after the first cardioembolic stroke, and of these recurrence events, 54% were also cardioembolic.^4^ According to the 2020 ESC guideline, early initiation of OAC and long‐term anticoagulant therapy are strongly recommended for NVAF patients experiencing AIS/transient ischemic attacks, as an important strategy to prevent recurrence of stroke/systemic embolism.^5^ However, previous studies showed, in China, OAC treatment rates among NVAF patients who were admitted to hospitals for new‐onset AIS were as low as 11%–19% at discharge,^6^, ^7^, ^8^, ^9^ indicating that, in the past decades, both neurologists and patients themselves did not attach enough importance to anticoagulant therapy. Recently, neurologists and cardiologists reach a consensus on post‐stroke anticoagulant therapy for AF patients,^10^ especially in the context of non‐vitamin K antagonist oral anticoagulants (NOACs) being observed with a lower risk of hemorrhage.^5^ However, it was unclear whether the adherence to anticoagulant therapy had been improved for NVAF patients who experienced new‐onset AIS, and whether there were any factors influencing doctors or patients to take OAC. The aims of our study are to investigate the proportion of NVAF patients who initiated OAC therapy after a new‐onset AIS and to explore its possible associated factors.

## 2 METHODS

### 2.1 Study population

The detailed design of China‐AF registry study has been previously described.^11^ From August 2011 to December 2018, a total of 25 512 AF patients were enrolled into China‐AF voluntarily and were followed up regularly every 6 months. In the current study, patients were recruited following the inclusion criteria: (1) age ≥ 18 years, and (2) diagnosed new‐onset nonfatal AIS during follow ups, and were excluded if: (1) patients were diagnosed with rheumatic mitral stenosis or having mitral valve prostheses, or (2) with serious chronic heart failure, or (3) with identified contraindications to anticoagulants. A total of 1085 new‐onset and nonfatal AIS patients that occurred during the follow ups were identified as our study population, with diagnoses based on brain computed tomography or magnetic resonance (referred to as index stroke). Written informed consent was obtained from each participant. The ethics committee of Beijing Anzhen Hospital approved the study.

### 2.2 Data collection

The following information were collected at baseline or each visit before the index stroke, including sociodemographic characteristics, lifestyles, AF types, medical history, history of radiofrequency ablation (RFA), results of laboratory tests. Three‐month‐peri‐stroke AF episodes were collected and diagnosed by 12‐lead ECG/24‐h Holter within 3 months before or after the index stroke. OAC usage information was collected at 3 months before the index stroke, and also at 3 months and each visit after the index stroke. Three‐month‐post‐stroke OAC usage was defined as the OAC usage at 3 months after the index stroke. The number of concomitant drugs indicated the total number of different types of drugs including statin, antiarrhythmic drugs, ventricular rate control drugs, angiotensin‐converting enzyme inhibitors or angiotensin receptor blockers, oral hypoglycemic drugs, antiplatelets. The CHA_2_DS_2_‐VASc score and HASBLED score were calculated for each patient.^5^, ^12^

### 2.3 Statistical analysis

Patients were classified into OAC group or non‐OAC group according to their 3‐month‐post‐stroke OAC usage. Baseline characteristics were reported as mean ± standard deviation (SD) for continuous variables and proportions for categorical variables, and compared using *t* test or *χ* ^2^ test between two groups. Multivariate logistic regression models were conducted to calculate the odds ratios (ORs) and their 95% confidence intervals (CIs) of factors, which might be associated with 3‐month‐post‐stroke OAC usage, such as age, sex, high‐reimbursement‐rate insurance, university graduated, persistent AF, interval since the first detection of AF, 3‐month‐peri‐stroke AF episodes, CHA_2_DS_2_‐VASc score, HASBLED score, history of RFA, pre‐stroke antiplatelet usage, pre‐stroke OAC usage and number of concomitant drugs.

A time‐to‐first‐event approach was used to explore the factors of non‐persistence of OAC among 3‐month‐post‐stroke OAC users within 2 years after index stroke. Time from the index stroke to stopping OAC therapy was defined as non‐persistence of OAC. Multivariate cox proportional hazards regression model was carried out to calculate the hazard ratios (HRs) and their 95% CIs of non‐persistence of OAC with previous mentioned factors. Adjusted survival curves of non‐persistence of OAC were plotted stratified by history of RFA.

All tests were two‐tailed and *p* values < .05 were considered statistically significant. All analyses were conducted using SAS statistical software version 9.4 (SAS Institute Inc.).

## 3 RESULTS

**Figure 1:**
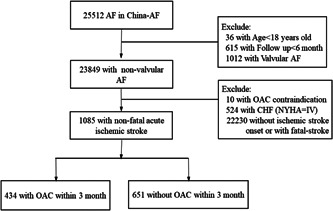
Flowchart of the study. AF, atrial fibrillation; CHF, chronic heart failure; OAC, oral anticoagulant

Among these 1085 nonfatal AIS patients (Figure 1), the mean age was 68.5 ± 10.4 years, and 43% were female. After the index stroke, 434 (40.0%) were 3‐month‐post‐stroke OAC users, and among the other 651 nonusers, 58.5% were using the antiplatelet agents.

Compared with the non‐OAC users, 3‐month‐post‐stroke OAC users were younger (67.6 ± 10.1 years vs. 69.1 ± 10.6 years, *p* = .017), with a higher proportion of university graduate (29.7% vs. 20.7%, *p* < .001), more likely to have high‐reimbursement‐rate insurance coverage (83.6% vs. 74.8%, *p* < .001), and had higher prevalence of persistent AF (47.5% vs. 36.9%, *p* < .001), embolism history (36.1% vs. 29.8%, *p* = .028), 3‐month‐peri‐stroke AF episodes (76.3% vs. 56.1%, *p* < .001) and higher proportion of pre‐stroke OAC usage (51.8% vs. 11.8%, *p* < .001), but lower mean HASBLED score (2.9 ± 0.8 vs. 3.4 ± 1.0, *p* < .001), lower mean number of concomitant drugs (1.9 ± 1.3 vs. 2.1 ± 1.5, *p* = .029) and much lower proportion of pre‐stroke antiplatelet usage (22.4% vs. 43.0%, *p* < .001) (Table 1).

**Table 1:** Baseline characteristics among NVAF patients with new‐onset AIS according to use of 3‐month‐post‐stroke OAC

Characteristics	All patients (*n* = 1085)	Non‐OAC (*n* = 651)	OAC (*n* = 434)	*P* value
Age (years)	68.5 ± 10.4	69.1 ± 10.6	67.6 ± 10.1	.017
Female	43.0% (467)	43.2% (281)	42.8% (186)	.920
Education				<.001
Elementary or below	226 (20.8%)	155 (23.8%)	71 (16.3%)	
Middle or high school	595 (54.8%)	361 (55.5%)	243 (53.9%)	
University or above	264 (24.3%)	135 (20.7%)	129 (29.7%)	
High‐reimbursement‐rate insurance	850 (78.3%)	487 (74.8%)	363 (83.6%)	<.001
BMI (kg/m^2^)	25.4 ± 3.4	25.2 ± 3.4	25.5 ± 3.3	.171
Smoking	162 (14.9%)	103 (15.8%)	59 (13.6%)	.311
Drinking	189 (17.4%)	112 (17.2%)	77 (17.7%)	.819
*Medical history*				
Radiofrequency ablation history	318 (29.3%)	204 (31.3%)	114 (26.3%)	.071
Persistent AF	446 (41.8%)	240 (36.9%)	206 (47.5%)	<.001
Hypertension	830 (76.0%)	502 (77.1%)	328 (75.6%)	.559
Chronic heart failure	198 (18.2%)	123 (18.9%)	75 (167.3%)	.499
Coronary heart disease	221 (20.4%)	145 (22.3%)	76 (17.5%)	.055
Embolism	351 (32.3%)	194 (29.8%)	157 (36.1%)	.028
Bleeding history	132 (12.2%)	80 (12.3%)	52 (12.0%)	.879
Diabetes mellites	388 (35.8%)	236 (36.3%)	152 (35.0%)	.679
Hyperlipidemia	517 (47.6%)	312 (47.9%)	205 (47.2%)	.823
Peptic ulcer	25 (2.7%)	16 (2.4%)	13 (3.0%)	.704
Interval since first detection of AF (years)	6.7 ± 7.0	6.4 ± 6.7	7.2 ± 7.4	.066
*Laboratory tests*				
eGFR <60, l/min·1.73 m^2^	38 (3.5%)	26 (4.0%)	12 (2.8%)	.315
CHA_2_DS_2_‐VASc score	5.1 ± 1.5	5.2 ± 1.5	5 ± 1.5	.057
HASBLED score	3.2 ± 1.0	3.4 ± 1	2.9 ± 0.8	<.001
3‐month‐peri‐stroke AF episodes	696 (64.1%)	365 (56.1%)	331 (76.3%)	<.001
*Treatment strategy*				
Antiplatelet	401 (37.0%)	381 (58.5%)	20 (4.6%)	<.001
ACE‐inhibitor/ARB	294 (30.7%)	204 (31.3%)	130 (30%)	.629
Statin	580 (53.4%)	317 (48.7%)	263 (60.6%)	<.001
Pre‐stroke antiplatelet usage	377 (34.7%)	280 (43.0%)	97 (22.4%)	<.001
Pre‐stroke OAC usage	302 (27.8%)	77 (11.8%)	225 (51.8%)	<.001
The number of clinics visit during stroke	0.9 ± 1.8	0.9 ± 1.6	0.9 ± 2.1	.911
Number of Concomitant drugs	2.0 ± 1.4	2.1 ± 1.5	1.9 ± 1.3	.029

Abbreviations: AF, atrial fibrillation; AIS, acute ischemic stroke; BMI, body mass index; NVAF, nonvalvular atrial fibrillation; OAC, oral anticoagulant.

From results of multivariate logistic regression models, 3‐month‐post‐stroke OAC usage was positively associated with high‐reimbursement‐rate insurance coverage (OR: 1.51, 95% CI: 1.03–2.22, *p* = .036), 3‐month‐peri‐stroke AF episodes (OR: 2.63, 95% CI: 1.88–3.69, *p* < .001), higher CHA_2_DS_2_‐VASc scores (OR for 1 score increase: 1.27, 95% CI: 1.08–1.48, *p* = .03) and pre‐stroke OAC usage (OR: 8.92, 95% CI: 6.01–13.23, *p* < .001), and was negatively associated with increased age (OR: 0.98, 95% CI: 0.96–1.00, *p* = .024), female (OR: 0.63, 95% CI: 0.44–0.90, *p* = .012) and higher modified HASBLED score (OR: 0.45, 95% CI: 0.37–0.55, *p* < .001) (Table 2).

**Table 2:** Associations of potential influencers with 3‐month‐post‐stroke OAC usage among NVAF patients with new‐onset AIS using multivariate logistic regression model

Variable	OR	95% CI	*P* value
Age (per 1‐year increase)	0.98	(0.96, 1.00)	.024
Female	0.63	(0.44, 0.90)	.012
High‐reimbursement‐rate insurance	1.51	(1.03, 2.22)	.036
University graduated	1.25	(0.89, 1.76)	.207
Interval since first detection of AF years, (per 1‐year increase)	1.01	(0.99, 1.03)	.576
Persistent AF	1.08	(0.80, 1.47)	.615
3‐month‐peri‐stroke AF episodes	2.63	(1.88, 3.69)	<.0001
CHA_2_DS_2_‐VASc score, (per 1‐score increase)	1.27	(1.08, 1.48)	.003
HASBLED score, (per 1‐score increase)	0.45	(0.37, 0.55)	<.0001
Radiofrequency ablation history	0.94	(0.66, 1.33)	.718
Pre‐stroke OAC usage	8.92	(6.01, 13.23)	<.0001
Pre‐stroke antiplatelet usage	1.27	(0.83, 1.94)	.269
Number of concomitant drugs			
0 type	Ref	Ref	Ref
1–2 types	0.76	(0.49, 1.19)	.227
≥3 types	0.66	(0.39, 1.13)	.130

Abbreviations: AF, atrial fibrillation; AIS, acute ischemic stroke; CI, confidence interval; NVAF, nonvalvular atrial fibrillation; OAC, oral anticoagulant; OR, odds ratio.

**Figure 2:**
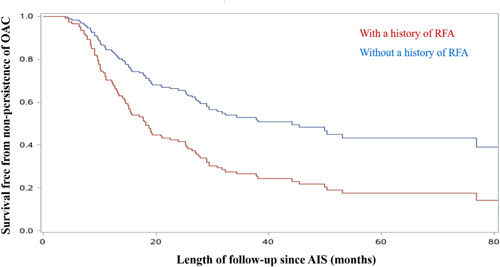
Adjusted survival curves for non‐persistence of OAC among 3‐month‐post‐stroke OAC users stratified by history of RFA. During adjustment, the covariates were set to: means of age, interval since the first detection of AF, CHA_2_DS_2_‐VASc score, and HASBLED score, and male, covering by low‐reimbursement‐rate, not highly educated, non‐persistent AF, with 0 type of concomitant drug, without 3‐month‐peri‐stroke AF episodes, without pre‐stroke OAC usage. AF, atrial fibrillation; AIS, acute ischemic stroke; NVAF, nonvalvular atrial fibrillation; OAC, oral anticoagulant; RFA, radiofrequency ablation

Among 434 3‐month‐post‐stroke OAC users, 396 patients were followed for an average of 26.9 months. And 168 (42.4%) were recorded discontinuing OAC therapy within 2 years after the index stroke, referred to as non‐persistence of OAC. From the multivariate Cox regression model, history of RFA (HR: 1.65, 95% CI: 1.16–2.35; *p* = .006) was positively associated with risk of non‐persistence of OAC, while 3‐month‐peri‐stroke AF recurrence (HR: 0.68, 95% CI: 0.37–0.99; *p* = .042) was associated with persistent OAC therapy (Table 3). Adjusted survival curves showed that, within 2 years after the index stroke of those 3‐month‐post‐stroke OAC users, persistence rate was lower in patients with history of RFA than those without RFA (Figure 2).

**Table 3:** Influencers associated with non‐persistence of OAC among NVAF patients with new‐onset AIS using multivariate Cox model

	OAC		
Variable	HR	95% CI	*P* value
Age (per 1‐year increase)	0.99	(0.97, 1.01)	.309
Female	0.88	(0.59, 1.31)	.514
High‐reimbursement‐rate insurance	0.98	(0.65, 1.49)	.929
Highly educated	1.18	(0.83, 1.69)	.355
Interval since first detection of AF, (per 1‐year increase)	1.00	(0.98, 1.02)	.773
Persistent AF	0.94	(0.68, 1.29)	.681
3‐month‐peri‐stroke AF episodes	0.68	(0.47, 0.99)	.042
CHA_2_DS_2_‐VASc score, (per 1‐score increase)	1.09	(0.92, 1.30)	.323
HASBLED score, (per 1‐score increase)	1.04	(0.84, 1.28)	.728
Radiofrequency ablation history	1.65	(1.16, 2.35)	.006
Pre‐stroke OAC usage	0.75	(0.54, 1.03)	.076
Number of concomitant drugs			
0 type	Ref	Ref	Ref
1–2 types	0.79	(0.51, 1.20)	.268
≥3 types	0.68	(0.42, 1.09)	.111

Abbreviations: AF, atrial fibrillation; AIS, acute ischemic stroke; CI, confidence interval; HR, hazard ratio; NVAF, nonvalvular atrial fibrillation; OAC, oral anticoagulant.

## 4 DISCUSSION

Among NVAF patients with new‐onset AIS in our study, the initiation rate of OAC within 3 months since index stroke remained low (40.0%). Possible factors that promote the early‐phase initiation of OAC after AIS were younger age, male, 3‐month‐peri‐stroke AF episodes, taking OAC before the index stroke, having high‐reimbursement‐rate insurance coverage, higher CHA_2_DS_2_‐VASc score, and lower HASBLED score. In addition, patients with a history of RFA were more likely to stop OAC therapy within 2‐year follow up.

### 4.1 OAC usage among NVAF patients with new‐onset AIS

Although anticoagulation is recommended to reduce risk of AF‐related stroke, OAC was underused in China.^6^, ^7^, ^8^, ^9^ A recent community‐based study reported that only 6.0% of AF patients with high stroke risk took OAC in China.^13^ From our AF registry, OAC therapy was initiated in 40% of patients within 3 months after the new‐onset AIS, which was higher than previous reports from the China NSR II study (19.4%),^8^ and the China QUEST study (11.0%),^7^ and was much higher than the general population (6.0% among AF patients with high stroke risk). ^13^ One reason might be that the China‐AF registry study is conducted by cardiologists, who usually pay more attention to OAC therapy,^14^ and patients who are enrolled in this registry could learn more knowledge of anticoagulation actively or passively; while the China NSR II study and the China QUEST study were conducted by neurologists, who are less trained on professional knowledge and skills of anticoagulant therapy. And the adherence to OAC therapy was even poorer in general population. However, the initiation rate of OAC in our study is much lower than that in developed countries (69.5% in Germany, 74.5% in RAF‐study, and 69.5% in Italy).^15^, ^16^, ^17^ In China, efforts are still needed to improve adherence of OAC therapy for NVAF patients with new‐onset AIS to reduce their risk of stroke recurrence.

### 4.2 Influencers on initiation of anticoagulant therapy after stroke

Under‐treatment of OAC is a common problem in low‐ and middle‐income countries,^18^, ^19^ for the limited medical resource, intolerable financial burden, and insufficient health education of patients and physicians.^20^ In our study, patients who were with high‐reimbursement‐rate insurance coverage, were more likely to initiate OAC treatment within 3 months after the new‐onset AIS, highlighting the necessity to lower down the out‐of‐pocket expenditure on medical treatment and strengthen health education on anticoagulant therapy among patients.^21^ In addition, we also observed patients who took OAC before were more likely to continue taking OAC after AIS, which indicated previous knowledge would strongly affect patients adherence to anticoagulant therapy.

From the evidence‐based guidelines, it is not advised to avoid OAC only because of higher bleeding risk, and the net clinical benefit of OAC is observed even greater among high‐bleeding‐risk patients.^5^ The ideal way is to eliminate or control the modifiable risk factors of bleeding before OAC therapy, that is, lower down the systolic blood pressure, improve renal/liver function, stop taking antiplatelet and abstain from alcohol, etc. For patients with unmodifiable risk factors (such as old age, having a medical history of stroke or bleeding), frequent follow‐up and monitoring would be needed,^22^, ^23^ and NOACs could be considered for its lower bleeding risk compared with warfarin.^24^ However, our study found the proportion of 3‐month‐post‐stroke OAC use was increased with higher risk of ischemic stroke (increasing CHA_2_DS_2_‐VASc score) but decreased with higher risk of bleeding (increasing HASBLED score), which showed a big gap between evidence‐based guidelines and clinical practice in China. Furthermore, the conflicts between doctors and patients which mostly caused by misunderstanding and communication inadequateness,^25^ and the higher bleeding risk among Asian patients during OAC therapy,^26^ may also postpone the doctors making decisions on anticoagulant therapy, especially for the patients who already have higher bleeding risk or have been treated with antiplatelet drugs.

In addition, our study showed that patients with 3‐month‐peri‐stroke AF episodes were more likely to take anticoagulants after stroke. The explanation could be that patients with more frequent recurrent AF would be more aware of the importance of stroke prevention, and it would be easier for them to accept the decision on anticoagulant therapy. So, we advise doctors to put more effort into letting patients know the necessity of anticoagulant therapy, even for the patients with lower AF burden.

### 4.3 Influencers on persistent use of OAC

Clinical guidelines recommended life‐long OAC therapy for patients with AF at increased stroke risk.^5^ A large population‐based cohort study indicated that patients with AF who discontinued OAC therapy had a significant twofold to threefold higher risk of ischemic stroke, compared with those who continued therapy,^27^ and another study showed OAC cessation was associated with excess risk of stroke.^28^ In our study, among patients who initiated anticoagulant therapy after AIS, 42.4% discontinued OAC therapy during a maximal follow‐up of 2 years, and we found that a history of RFA procedure might be an independent factor associated with stopping anticoagulant therapy. Although RFA could reduce AF burden,^29^ and a previous study based on China‐AF registry observed that the thromboembolic risk after successful RFA was low in non‐OAC patients and discontinue anticoagulation might be safe,^30^ the evidence is still scanty whether patients could quit OAC therapy after the RFA procedure. According to the Guidelines, CHA_2_DS_2_‐VASc scores should be considered rather than AF burden, and AF patients with higher stroke risk should continually take OAC no matter they had RFA procedure or not.^5^ However, further research is needed to provide more evidence on anticoagulation therapy after RFA.

### 4.4 Limitations

First, despite this is a multicenter study, the selection bias was still difficult to avoid. Second, NOACs were not available until 2013 in China, and were not covered by social health insurance until 2019. Among 434 3‐month‐post‐stroke OAC users, 190 (43.8%) were taking NOACs, and the sample size was quite small to compare the difference of persistence between NOACs and warfarin. Third, the detailed information on whether the doctors, usually neurologists, prescribed OAC for new‐onset AIS patients during hospitalization was not collected, our data was not adequate to evaluate the neurologists' adherence to guidelines for the management of AF patients. Fourth, the duration between index stroke and the initiation time of OAC was not recorded precisely, and the time lag between the admission date and the follow‐up date after the stroke was used as substitutes. Fifth, whether the new‐onset AIS were cardioembolic was not able to identified in this study, so, we could not answer if post‐stroke anti‐thrombotic choice was influenced by the type of stroke. Additionally, although we performed multivariable models to adjusted measured confounders, there may still be unmeasured confounding that remains.

## 5 CONCLUSION

In China, the proportion of NVAF patients who initiated OAC therapy after new‐onset AIS was still low. Older age, female, higher bleeding risk, lacking knowledge of anticoagulant therapy and higher economic burden might be factors that hinder the initiation of OAC therapy, and those OAC users with a history of RFA procedure were more likely to stop taking anticoagulants. Our findings imply that it is still necessary to train Chinese doctors to acquire more professional knowledge and skills of anticoagulant therapy, and also to make more targeted efforts to improve AF patients' awareness of why and how to take anticoagulants properly.

## CONFLICT OF INTERESTS

Chang‐Sheng Ma has received honoraria from Bristol‐Myers Squibb, Pfizer, Johnson & Johnson, Boehringer‐Ingelheim, and Bayer for giving lectures. Jian‐Zeng Dong has received honoraria from Johnson & Johnson for giving lectures. The remaining authors declare that there are no conflict of interests.

## Data Availability

The data that support the findings of this study are available on request from the corresponding author. The data are not publicly available due to privacy or ethical restrictions.
